# USDA animal genomics program: the view from the chicken coop

**DOI:** 10.1186/1471-2164-10-S2-S1

**Published:** 2009-07-14

**Authors:** Janet E Fulton

**Affiliations:** 1 Hy-Line International, PO Box 310, Dallas Center, IA 50063, USA

## Abstract

In 2007, the USDA Animal Genomics Strategic Planning Task Force prepared a Blueprint to direct national needs for future research, education, and extension efforts in agricultural animal genomics. This plan is entitled "Blueprint for USDA Efforts in Agricultural Animal Genomics 2008–2017". The Blueprint is reviewed from the perspective of a molecular biologist working within the poultry breeding industry. The diverse species used in animal agriculture require different tools, resources and technologies for their improvement. The specific requirements for chickens are described in this report.

## Background

The last few years have been exciting times in the chicken genetics community. We have a sequence of the chicken genome [1], we have 2.8 million single nucleotide polymorphisms (SNPs), the chicken has been recognized as a biomedical model organism by NIH and we have easy access to many tools to analyze chicken genomic information. These tools, resources and philosophies have all opened many more avenues for poultry research than were available just 10 years ago. Genome sequence is now available for cow and will soon be available for pig and turkey.

In 2007, the USDA developed a "Blueprint for USDA Efforts in Agricultural Animal Genomics 2008–2017" [2] (hereafter referred to as the "Blueprint"). The purpose of the Blueprint was to define USDA research priorities with the goal of advancing the application of genomic technologies to enhance animal agriculture.

The Blueprint attempts to set goals for the next decade for all animal agricultural species. This includes the very diverse species used for agricultural production, covering cattle, sheep, pigs, chickens, turkeys, and multiple species of fish, shrimp and oysters. The Blueprint is divided into 3 sections: 1 Science to Practice – a discussion of the technologies currently available for genomics studies; 2 Discovery Science – a description of the knowledge gaps that need to be filled and 3 Infrastructure – a description of the various tools and resources that are needed.

This Blueprint articulates its important points with a significant bias towards the cattle industry. However, each agricultural animal industry has different issues, concerns and perspectives. This review examines the Blueprint from the perspective of a molecular biologist working within the poultry breeding industry. The following comments express the unique and specific needs of the poultry industry for the three sections of the Blueprint.

## Science to practice

The primary focus of the "Science to Practice" section is to increase the rate of genetic improvement in breeding stock. Quantitative genetics was introduced into animal improvement plans during the early 1950's and has been responsible for tremendous increases in animal production efficiency over the past half century. Genomics information is rapidly increasing for the major animal production species, and its application is logically viewed as the next giant step in animal improvement technologies.

### Whole genome selection

Whole genome selection (WGS) has the potential to be a very valuable tool, and this potential can be tested for those species with sequence information and large numbers of genetic markers. WGS is being explored through two newly announced grants of $2.5 million each, covering chicken and cattle. The value of WGS is seen as increasing the rate of selection while simultaneously decreasing progeny testing costs and allowing selection progress to be monitored. This is still very theoretical research, and there must be some caution applied to not 'oversell' the possible results. Yes, the potential application results could be very high, but the cost of the testing must also be considered. The value of one breeding chicken is considerably less than that of one bull or even one cow. Recent costs of SNP typing are estimated at $300–$400 for an individual. Since the number of birds within one breeding line can range from 5,000 to 8,000, the cost of genotyping one line would be $1.5 to $3.1 million. Trait measurements would then need to be added to the overall cost. Also, since each commercial product is produced from 4 parental lines, the total cost would have to be multiplied by a factor of 4. The generation interval for chickens is relatively short (one year). WGS can help to shorten that time to 6 months. This is very useful research, with high application value and definitely belongs within the realm of technology research within the USDA. However, the different industry structures and reproductive rates must be kept in perspective for each species.

### Prediction of genetic merit

The USDA Blueprint proposes to use genome-based information plus phenotype data to predict the best individuals. This requires integration of genomic information with phenotype data and incorporation within existing quantitative genetics theory. This is very important for all agriculture animal species. This theory is likely to have cross-species applications. USDA proposes to develop standardized trait definitions and recording systems. Within each poultry breeding company, phenotype definitions and measurements are already standardized. These traits and their measurement protocols are the core principles that each company uses to increase the productivity of their products. These are considered proprietary as they maintain each company's competitive advantage.

### Integration of genomic data

Appropriate species-specific tools are needed to allow for integration of genomics information into the existing selection systems used by breeding companies.

### Precision management

According to the Blueprint there is considerable interest within the livestock sector to identify the genotype of an individual animal or specific breed or strain so that the optimal production environment for that animal can then be determined, thus optimizing animal well-being and production efficiency. This philosophy is completely opposite to that used for commercial egg-laying chickens. For many generations, poultry breeders have selected for animals that can exhibit maximum adaptability or that can perform in multiple environments. This is accomplished through the use of very large progeny testing programs in multiple international locations. These field tests are conducted under different environmental stresses, feed quality, disease status, management conditions etc, all with the goal of producing animals that are adaptable to a variety of production environments throughout the world.

### Parentage identity and traceability

Parentage identity and traceability of meat products are of major concern for the cattle industry, in light of concerns with pedigree errors and Bovine spongiform encephalopathy (BSE). Within Hy-Line International, elite line identity has been possible for over three decades, first through the use of blood-types and more recently with microsatellite data. Each of the elite stocks can be identified and so can their subsequent commercial products. Traceability of individual meat/egg sources is more problematic. With approximately 5.5 million layer parents and 0.5 billion broiler parents in turn producing the billions of commercial production layers world-wide, traceability of individuals may not be possible. These parent stocks are at least four generations away from elite animals selected individually in pedigreed populations. Once again, the cost of individual animal typing in contrast to the value of the animal may make genetic tracing of parental poultry stock prohibitive.

## Discovery science

Discovery Science attempts to identify those critical gaps in knowledge that need to be covered before genome-enabling technologies can be fully applied to animal production agriculture.

### Gene identity for production traits

There has been considerable research in the past decade looking for genomic regions that harbour genes that influence production trait quantitative genes. Many quantitative trait loci (QTL) have been found, but few actual genes or responsible genetic variants have been identified. Within poultry, most of these studies have been done utilizing diverse breed crosses, such as broiler × layer. Yet for practical industry application, we need to identify QTLs that are segregating within a line. We would expect to find QTLs for traits that have major differences between broilers and layers, (e.g. growth rate, breast yield, egg production), but these major genetic influences are probably already fixed within the extensively selected elite lines. It is the within line variation that is of the most use, and very few studies have been examined for that level of variation. It is also important that the traits examined in QTL studies are relevant to production traits, and these can differ greatly between broilers and layers. Many of the chicken chromosomes are not well covered in the existing QTL studies as genetic markers in these areas are limited or non-existent. More QTL studies are definitely needed. Information on which genetic regions influence traits, coupled with complete genome sequence information will be of great benefit in moving us toward identification of production related genes and understanding of their role in influencing traits.

The current methodology for identifying genes involves comparisons with known information from other species. There is considerable information on mammalian genes, due to the vast amount of information from human and mouse genomes. However, since the chicken is currently the only avian species with genome information, it is much more difficult to interpolate from comparative genomics. Recent efforts are underway to develop applicable gene annotation terms and tools for poultry [3].

### Systems biology

The Blueprint recognizes that genes do not function in an isolated system, but rather within an entire organism. Thus, expression within the context of an entire individual needs to be understood. Gene expression can be examined in diverse animal populations, and technologies such as transgenesis, gene knock-outs or RNA interference can be used.

Unfortunately, the differences between birds and mammals make current mammalian developed technologies very difficult to apply in chickens. There are some methodologies available, but they are expensive, time-consuming, and not yet practical for routine utilization for gene function studies. A successful USDA/ARS transgenesis program was terminated several years ago due to insufficient funds. RNAi research is very new, but because of inadequate funding in the US, research on this technology has defaulted to other countries. The US needs more research to develop these technologies for avian species. Emphasis over the past few years within USDA has been towards research with direct poultry industry application. This has decreased available funds for basic research and development of novel technologies. However, novel technologies must be developed before they can be successfully implemented in commercial poultry

### Genetic variation

Regrettably, most of the phenotypic diversity in non-commercial poultry has been lost due to lack of long-term funding for support of resource populations over the past five decades. While the US used to have the most diverse experimental poultry populations, phenotype variants, and specially selected strains, that is no longer the situation. A few very interesting studies have been done recently comparing the growth characteristics of broilers representing the 1970's and modern broilers [4]. Similar studies cannot be done for layers because the equivalent stocks were eliminated in the early 1990's. Very few locations have non-commercial derived poultry stocks left. There are few pure breeds left within the university systems, and many of those available from hobbyists have unknown disease status, as well as unknown pedigrees.

### Host-microbial interactions

Agriculture production does not occur in a sterile environment. The interaction of animals with their environment includes relationships with numerous microbial genomes including harmful pathogenic organisms as well as commensal gut microbial communities. As the Blueprint states, host-microbial interaction studies require systems biology approaches with interdisciplinary teams of scientists. At the time of writing, an excellent USDA/ARS interdisciplinary team that deals with host-microbial interactions (ADOL, East Lansing, MI) is slated to be closed due to lack of funds in the 2008–2009 budget. The Blueprint suggests that these approaches are needed, yet at the same time, budgets are insufficient to maintain existing, very effective programs.

Microbial genome sequences are being produced. Generally these are for pathogens of concern for human health. However, better information on genomes of microbes affecting animal health will enhance animal production, which in the long term will enhance human health. Thus, sequence information is needed for microbial genomes relevant to animal production.

## Infrastructure

Within the Blueprint, infrastructure is defined as the genomics tools and resources that are needed in agricultural animal genomics research, plus the skilled work force required to utilize these tools. The four Blueprint sections are genomic tools, comprehensive databases with statistical and bioinformatics tools, centralized animal resource populations, and education and training of students. The Blueprint clearly states that physical buildings (i.e. bricks and mortar) are not needed.

### Genomics tools

The Blueprint targets the availability of a 10 fold (or greater) coverage of the genome of all top tier species, (chicken, swine, cattle). The chicken genome sequence currently has 6.6× coverage, which is the coverage level suggested for the next tier of economically important species (e.g. catfish, sheep, tilapia). However, the current chicken sequence is still incomplete. It is missing nine microchromosomes, plus most of chromosome 16 which is of particular interest as it contains the chicken MHC. In addition, 17,000 contigs are unanchored, which represents a significant proportion of the chicken genome and may well include the missing nine microchromosomes.

At the same time that the first build of the genome sequence was released, 2.8 million SNPs were released by the Beijing Genome Institute [5]. These SNPs were developed from a 0.3 × coverage of three breeds of chickens: Chinese Silkie, commercial broiler, and a laboratory Leghorn line. The identified polymorphic differences between these three breeds and the jungle fowl were used to generate the genome sequence. Half of all commercial egg production is provided by brown egg layer stocks. These utilize different breeds and thus their SNP polymorphisms are probably very under-represented in this first SNP set. The laboratory Leghorn line was somewhat inbred, and may also under-represent the SNP polymorphisms within the commercial Leghorn lines. We need to determine if the current 2.8 million SNPs have universal value for all commercial type birds, as well as other non-tested breeds.

### National, comprehensive databases and statistical and bioinformatics tools

Each poultry breeding company has its own in-house comprehensive databases that store information for all of their lines. These databases contain extensive information on thousands of individuals for multiple generations and multiple phenotypes. This is the primary resource used by quantitative geneticists for selection and subsequent genetic improvement each generation. Standardized traits and selection indices are already in place within each breeding company. It is doubtful that breeding companies will freely share this type of information as this is what defines the commercial products of each company, and is how each company derives its' success and distinguishes itself from the competition

Chicken genome sequence information is currently being maintained and curated by the National Center for Biotechnology Information (NCBI). NCBI should continue to maintain and curate these resources, particularly the chicken genome. It is a model species and the most studied avian species. USDA has funded database development in the past, however past experiences show that these databases rapidly become unusable once grants have expired as there are no long-term funds for maintenance and upgrading. New information is not added, and computer errors accumulate, resulting in non-accessible information. One central repository of information with long-term funding commitments makes more sense. Development of software that can be used to analyze unique populations based on agriculture animal species mating schemes is of critical importance.

### Centralized animal genetic resource populations

The production and maintenance of specialized animal genetic resource populations is very expensive. The Blueprint proposes to develop a centralized facility to preserve unique experimental populations, which can then be genotyped and phenotyped and subsequently made available to the genomics community (both agricultural and biomedical). As previously mentioned, there has been a considerable decrease of what was once a significant national collection of poultry germplasm. This is likely to continue as funds decrease at the state level. Grants provide only short-term support, yet maintenance of poultry diversity requires continuous funding. The decline of diversity has been outlined by [6] and again by [7]. Since 2004, there has been even more loss of avian genetic diversity. This loss includes the elimination of chromosome rearrangements from the University of Wisconsin, MHC congenic recombinant strains from the University of New Hampshire and 9 developmental mutants from the University of Connecticut will be eliminated in 2008 http://www.canr.uconn.edu/ansci/poultry/website.htm. The University of California, Davis has the largest collection of poultry genetic diversity left in the US. This stock is under constant threat of being eliminated due to ever decreasing funds http://animalscience.ucdavis.edu/AvianResources/.

Is a centralized facility the best option for poultry? Having resources at only one location increases the risk of disease and natural disaster vulnerability and susceptibility to local budgetary limitations. For example, during the Exotic Newcastle Disease (END) outbreak in California in 2002, there was concern that the UC Davis stocks might need to be destroyed since the depopulation of poultry stocks in END affected areas was underway.

### Education and training

There has been increased effort to train genomics students, but this has been at the expense of basic sciences. There has been a steady decline in the number of universities offering training in poultry sciences over the past five decades despite increasing poultry consumption and increasing value of the industry [8,9]. Industry is having an increasingly more difficult time finding scientists at all levels with poultry knowledge and experience. Research in genomics is very important, but we still need information on the basic sciences, such as nutrition, management, physiology, immunology, so that we can integrate genomics and basic information. The significance of appropriate funding for agricultural research for the future well-being of the industry was discussed by Scanes [10].

The Blueprint categorically states that bricks and mortar are not needed. This is absolutely not the case for poultry. Because poultry cannot be raised on pasture, they need buildings and facilities. The main USDA poultry genomics facility (ADOL) was built in 1939, with laboratory building additions in the early 1970's. The wooden poultry houses there are between 50 and 68 years of age. When research was needed in the mid 1990's, the poultry industry had to supply funds to purchase cages that were appropriate to hold modern broiler birds. The birds have changed, management has changed, but unfortunately the facilities have not, and they are no longer adequate for the research needed. In the USDA, there is a lack of scientists who possess poultry genomics research expertise, and there are no modern poultry housing facilities that can house the number of birds needed for genomics research.

## Summary

The Blueprint is incomplete as it misses many relevant areas for poultry. Poultry are not mammals, thus many tools developed for mammals are not useable. Genomics can only be applied in conjunction with basic science information for poultry. These basic science resources are rapidly decreasing, particularly for poultry species. The need for these basic resources is not recognized within the Blueprint.

Funding levels for research with poultry species do not keep up with the needs. Grant success rates are so low that scientists are discouraged from doing basic research with poultry species. University departments are reluctant to hire new scientists in areas in which funding is poor, thus faculty hires are based on likelihood of grant success, not on need for teaching or research. USDA/ARS cannot currently fund ARS scientists at the minimum support level. USDA/ARS genomics programs are being closed due to lack of funding resulting in an inconsistent message from USDA regarding the importance of genomics for poultry.

The needed areas for poultry genomics research as viewed from within the poultry industry are summarized below.

1. Science to practice

a. Integration of genomic information with existing selection methods is needed and should be approached from both the theoretical and practical aspects.

b. Genotyping costs need to reflect the value of the individual animal, which can vary considerably depending on its place in the breeding/production pyramid.

2. Discovery Science

a. Studies are needed that identify within line variation.

b. Identification of genes and genetic variants within QTL regions, along with appropriate annotation is needed.

c. QTL scans with coverage of all chromosomes are needed.

d. Fundamental tools employing RNAi or transgenesis must be developed.

e. Microbial genomics of pathogens and commensal organisms relevant to poultry should also be included.

3. Infrastructure

a. The recommended 10 × genome coverage is needed.

b. Complete genome sequence (including 10 missing chromosomes) must be available.

c. Curation of the genome needs long-term support.

d. A mechanism must be developed for maintaining genetic diversity, especially for non-commercial stocks. Existing germplasm must be maintained until we have cryopreservation techniques available.

e. Modern animal facilities that can hold contemporary birds with contemporary management methods are needed.

f. Education and training must also include basic sciences such as nutrition, physiology, management.

## Competing interests

The author declares that they have no competing interests.

## Authors' contributions

JEF conducted the review of the BluePrint and wrote the paper.

## References

[B1] Hillier L International Chicken Genome Sequencing consortium Sequence and comparative analysis of the chicken genome provide unique perspectives on vertebrate evolution Nature 2004 432 695 716 10.1038/nature03154 15592404

[B2] USDA Animal Genomics Strategic Planning Task Force Blueprint for USDA Efforts in Agricultural Animal Genomics 2008–2017 2007

[B3] The AgBase Database http://www.agbase.msstate.edu/

[B4] Havenstein GB Ferket PR Qureshi MA Growth, livability, and feed conversion of 1957 versus 2001 broilers when fed representative 1957 and 2001 broiler diets Poultry Science 2003 82 1500 1508 14601725 10.1093/ps/82.10.1500

[B5] Wong G 114 co-authors: International Chicken Genome Sequencing Consortium A genetic variation map for chicken with 2.8 million single-nucleotide polymorphisms Nature 2004 432 717 722 15592405 10.1038/nature03156 PMC2263125

[B6] Fulton JE Delany ME Poultry Genetic Resources – operation rescue needed Science 2003 300 1667 1668 10.1126/science.1085407 12805523

[B7] Miller MM Genome news highlights loss of chicken strains Nature (Correspondence) 2004 432 799 10.1038/432799a 15602523

[B8] Pardue SL Educational opportunities and challenges in poultry science: Impact of resource allocation and industry needs Poultry Science 1997 76 938 943 9200227 10.1093/ps/76.7.938

[B9] Beck MM Status of poultry science departments and poultry research within combined departments Poultry Science 1992 71 1328 1331

[B10] Scanes CG The case for funding agricultural research Poultry Science 2007 86 2483 2782 10.3382/ps.2007-86-12-2483 18029792

